# The 2019–2020 volcanic eruption of Late’iki (Metis Shoal), Tonga

**DOI:** 10.1038/s41598-022-11133-8

**Published:** 2022-05-06

**Authors:** I. A. Yeo, I. M. McIntosh, S. E. Bryan, K. Tani, M. Dunbabin, D. Metz, P. C. Collins, K. Stone, M. S. Manu

**Affiliations:** 1 National Oceanography Centre Southampton, Southampton, UK; 2 Japan Agency for Marine-Earth Science and Technology (JAMSTEC), Tokyo, Japan; 3 School of Earth and Atmospheric Sciences, Queensland University of Technology, Brisbane, Australia; 4 National Museum of Nature and Science (Kahaku), Tokyo, Japan; 5 School of Electrical Engineering and Robotics, Queensland University of Technology, Brisbane, Australia; 6 Queens University Belfast, Belfast, UK; 7 Vava’u Environmental Protection Association (VEPA), Vava’u, Tonga; 8 Natural Resources Division, Tonga Ministry of Lands, Survey and Natural Resources, Nuku′alofa, Tonga

**Keywords:** Geology, Volcanology, Geochemistry

## Abstract

Late’iki (previously known as Metis Shoal) is a highly active volcano in the Tofua arc with at least four temporary island-building eruptions and one submarine eruption in the last 55 years. The most recent eruption, commencing in October 2019, resulted in lava effusion and subsequent phreatic explosions, the construction of a short-lived island that was quickly eroded by wave action and possibly further phreatic activity that continued into January 2020. The two-pyroxene dacite from the 2019 eruption is similar to the 1967/8 eruptions suggesting the magma is residual from earlier eruptions and has not undergone further differentiation in the last 50 years. New observations of the 2019 eruption site confirm the lava-dominant character of the volcano summit but a thin veneer of wave-reworked, finely fragmented lava material remains that is interpreted to have been produced by phreatic explosions from hot rock-water interactions during the effusive eruption. A notable absence of quench-fragmented hyaloclastite breccias suggests that non-explosive quench fragmentation processes were minimal at these shallow depths or that hyaloclastite debris has resedimented to greater depths beyond our summit survey area.

## Introduction

**Figure 1:**
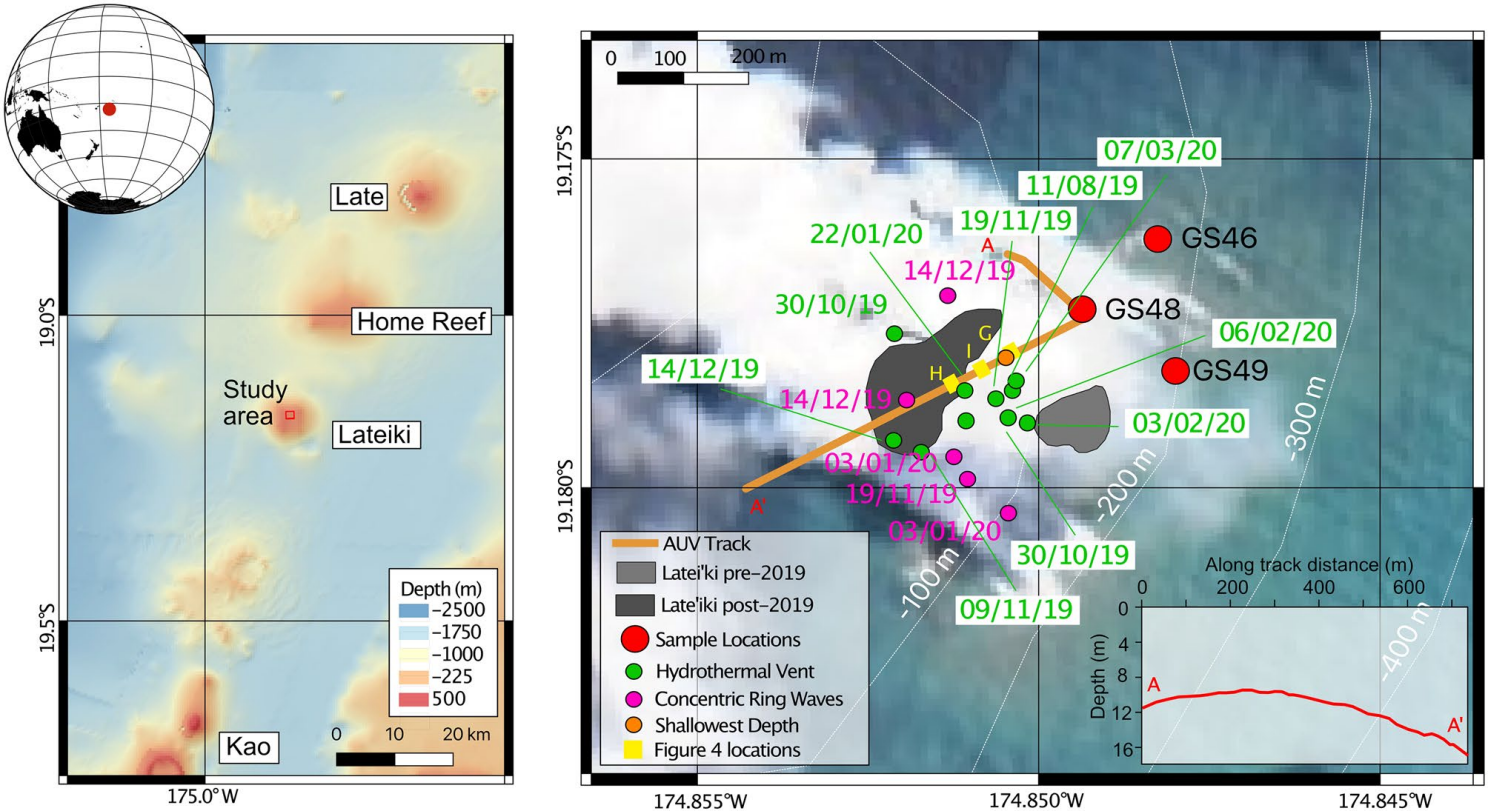
Late’iki location. Left hand panel shows the study area in its regional context. The bathymetry is reproduced from GEBCO Sheet G.08 compiled by R.L. Fisher of the Scripps Institution of Oceanography and extracted from the GEBCO Digital Atlas^58^. The right hand panel shows Sentinel-2 satellite imagery of the eruption site on the 20th October 2019^59^. Sampling locations and AUV track are plotted alongside locations of concentring ring waves and hydrothermal venting mapped from satellite imagery. The approximate locations of the photo mosaics from Fig. 4 are shown in yellow. The inset profile shows the water depths along the AUV track over the summit of Late’iki post eruption in February 2020.

The ~ 800 km long Tofua volcanic arc has a relatively high eruption frequency (~ 3 per decade, with two known eruptions in 2019). It contains 20 known active volcanoes, including 13 that are submarine, which erupt basaltic andesite, andesite and dacite magmas both effusively and explosively^1–5^. Dacititic products were considered relatively uncommon and largely restricted to subaerial Fonulaei^2,3^; however, pumice rafts from submarine eruptions^6–8^ and dredged lavas from some submarine edifices are also dacitic^9,10^. Consequently, the volume of dacite magma erupted in the Tofua arc has been underrepresented due to the few available subaerial deposits. The ephemeral island-producing dacitic volcano of Late’iki (previously known as Metis Shoal), centrally located in the Tofua arc at 19.17° S and 174.85° W (Fig. 1), is thus an important target for understanding both the geochemistry and eruptive behaviour of these volcanoes.

### Historic eruptions of Late’iki

Late’iki is a shallow to emergent submarine volcano (summit currently < 10 m below sea level) and erupts regularly, with recorded eruptions in 1781, 1851, 1858, 1878, 1886, 1967–8, 1979, 1990–1, 1995 and 2019^11,12^, each characterised by low explosivity and lava extrusion. Six temporary islands formed from the 1781–1995 eruptions; of these, the 1967–8 and 1979 eruptions also produced floating pumiceous material, and one eruption in 1990–1 is thought to have been entirely submarine and also produced floating pumice^12^. In contrast to subplinian to plinian pumice raft-producing eruptions from the nearby Home Reef and 0403-091 (also known as Volcano F) submarine volcanoes, where the main eruption phase lasts < 1 day^8,13,14^, eruptions at Late’iki last several weeks^15–17^. The earliest well-documented eruption (1967) began from a submarine vent with lava fountains and tephra ejections reaching 1.2 km altitude^18^, producing a small island 800 m long and ~ 15 m high that was removed by wave action within ~ 6 weeks^15^. Lavas were poorly—moderately vesicular^19^ dacites susceptible to breakage and wave erosion^15^. In May 1979 (possibly beginning earlier), Late’iki erupted again producing an island and explosions that ejected material ~ 150 m above the ocean with ash and steam reaching 1.5 km^20^. This eruption is presumed to be the source of an floating pumice raft observed NW of Late island in May 1979, which reached Fiji and the Solomon Islands^21^. A July 1979 survey by RV Balikula revealed a rapidly eroding island 300 × 120 m and ~ 15 m high, located approximately 1 km E of the former 1968 island^22^, with a tephra rampart marked by drainage gullies that was removed by early October 1979^23^. In contrast, the next island-forming eruption (1995) produced an emergent lava dome. An early video recording by a citizen scientist (Allan Bowe) showed explosive activity continuing during lava extrusion with ash eruptions from two vents ejecting material to ~ 500 m above sea level and steam rising to 2 km^17,24^. The maximum island size was ~ 300 × 250 m and ~ 50 m high; it endured until its destruction in the 2019 eruption^20^.

**Figure 2:**
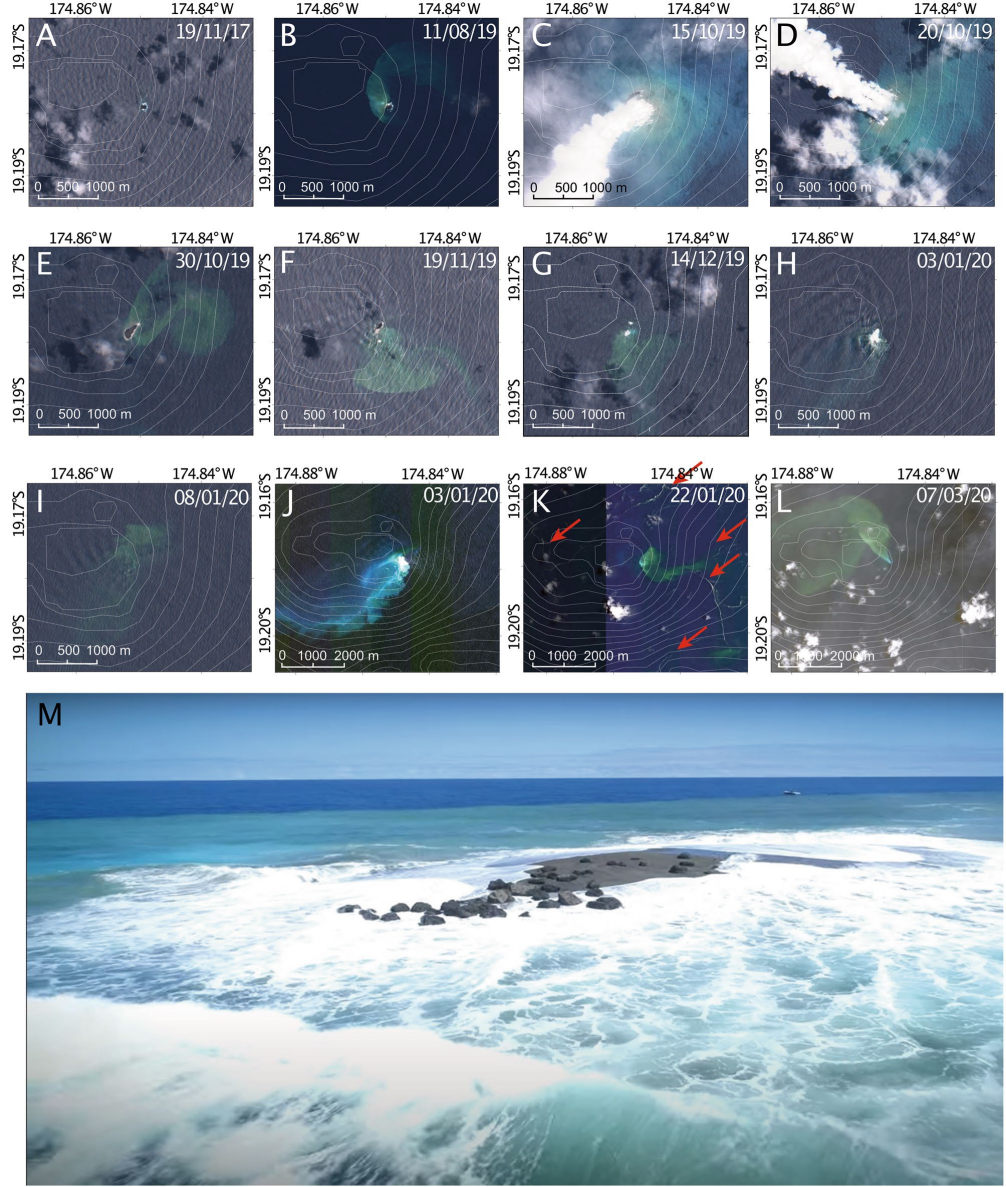
Satellite imagery of the 2019–2020 eruption phases. (**A**–**I**) Sentinel imagery^59^ showing the same region before, during and after the October 2019 eruption. Superimposed 100 m contours are from the GMRT grid^60^ but are not very accurate in this area and are for orientation only. (**J**–**L**) MAXAR WorldView-2 and WorldView-3 satellite data of the region showing key events^53^. Contours are the same as in panels (**A**–**I**) but the maps are shown at a different scale. Red arrows in panel K show floating material in the water surrounding the volcano. (**M**) Drone view of the remains of New Late’iki island on December 3 (taken by Darren Rice of Matafonua Lodge). The island has been reduced to sea level, being continually wave swept and comprises outsized metre-sized blocks of dacitic lava resting on lapilli to ash sized fragmented lava material generated by phreatic explosions. The island is surrounded by an extensive hydrothermal plume.

The 2019 eruption of Late’iki was first detected by a ship at 0800 on 14 October and reported by the Tongan Ministry of Lands and Natural Resources later the same day (Appendix X.1). The eruption was confirmed by NASA MODIS data on 13 October (UTC), imaged in higher resolution by the Sentinel-2 satellite on 15 October (Fig. 2C; Appendix X.2), and continued until at least 20 October (Fig. 2D), with steam visible in MODIS imagery until at least 23 October (see Plank et al.^16^ for more details). A new island was formed 150 m WNW of the former 1995 Late’iki Island (Fig. 1). This 2019 New Late’iki Island existed from 15 October, reaching a maximum size of ~ 21,000 m^2^ on 30 October (Fig. 2E) before eroding quickly (960 m^2^ per day) until 19 November (Fig. 2F) and then more slowly (124 m^2^ per day) until 14 December (Fig. 2G), when the island was only discernible from wave breaks. By the 3 January the island was totally submerged. Amateur drone footage on 3 December 2019 revealed a small wave-swept island at sea level marked by scattered metre-sized lava blocks (Fig. 2M). Rapid erosion, relative to the ~ 24 year duration of the 1995 island, was interpreted to result from island construction by easily erodible pyroclastic debris^16^.

In this contribution we present high-resolution satellite imagery alongside seafloor imagery and sampling of the post-eruptive summit collected in February 2020 (see “Methods”) to tie remote observations to physical processes.

## Results

### Remote observations

#### Formation and lifespan of New Late’iki island

**Figure 3:**
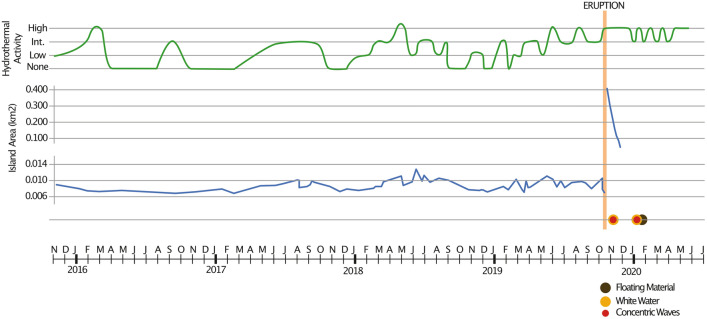
Satellite observations of hydrothermal venting intensity, island area, white water caused by phreatic activity, concentric ring waves and floating material plotted against time in the period before, during and after the 2019 eruption. Island areas are plotted on two separate scales as New Late’iki was considerably larger than the pre-eruption island.

The 1995 Late’iki Island is visible in Sentinel-2 imagery collected prior to the 2019 eruption (e.g. Fig. 2A,B). Although smaller than its 1995 maximum size the 110 × 85 m island shows little change in size or shape over a period of several years (Fig. 3), supporting the interpretation that the lava was more resistant to wave erosion than previous islands of more fragmented material^16^. NASA MODIS and Sentinel-2 imagery of the 2019 eruption showed a large steam plume obscuring the original island (Fig. 2C,D) and a new, elongate edifice built 150 m to the NW; the eastern edge of this island appeared to retreat while the western end grew during the eruption, finally forming the teardrop-shaped New Late’iki island by 30 October^17^ (Fig. 2E) which, despite being larger than the 1995 island, rapidly decreased in size. Drone footage on 3 December shows a low-relief wave-swept mound already reduced to sea level (Fig. 2M) and clear Sentinel-2 imagery from 3 and 8 January (Fig. 2H,I) confirm the island was submerged by early January 2020.

#### Evidence for post-October 2019 activity

After the disappearance of the steam plume on 23 October (Fig. 2C,D), Late’iki seemingly became quiescent. However, satellite imagery suggests several incidences of ongoing activity during the period between 23 October and 23 January 2020. These events are much smaller than the main eruptive phase and short-lived, but suggest minor extrusion and/or magma-water interaction continuing at the vent site following the main eruptive phase. There are three such events picked up in the satellite imagery, on 19 November, 3 January and 22 January, though others may have occurred but not been imaged. During these events two or more of the following phenomena are observed: (1) white water, not associated with New Late’iki Island (19 November, 3 January); (2) concentric ring waves, not observed at other times (19 November, 3 January); (3) stringers of floating material in the water—these mostly follow the dispersal of the hydrothermal plume, suggesting dispersal by currents, though several are also observed elsewhere, likely due to the dynamic nature of the currents in this region and a prevailing southerly to westerly wind direction during the period (22 January); (4) steam plumes rising from the vent area of the volcano (3 January); and (5) high levels of seawater discolouration (19 November, 3 January, 22 January). All these events occur within 300 m of New Late’iki Island and in the vent region for the main October 2019 eruptive phase.

#### Hydrothermal activity

Late’iki has had persistent hydrothermal activity for at least the last 5 years (Figs. 2, 3). The visible extent of the hydrothermal plume depends somewhat on the predominant ocean current direction and strength at the time, but different intensities of venting are discernible, including periods with no observed hydrothermal outflow (Fig. 2). We identify activity levels of zero to high activity (see “Methods”) with which to qualitatively examine the robustness of hydrothermal outflow before and after the 2019 eruption (Fig. 3).

Intermittent hydrothermal venting is observed in the earliest available Sentinel-2 imagery in November 2015 (Figs. 1, 2B). Three periods of hydrothermal outflow followed by periods of quiescence occurred between January 2016 and January 2018. From February 2019 to the eruption there is an increase in venting intensity that remains at consistently high levels in the weeks preceding the eruption.

During the October 2019 eruption period, discoloured water extends over 1.5 km all around the newly-formed island. After the end of the main eruptive phase on 30 October (Fig. 2E), hydrothermal activity remained high, apparently sourced from two locations on the NW and SE of the island, 100 m WSW of the pre-eruption hydrothermal site (Fig. 1). On 9 November (Appendix X.1), hydrothermal activity increased again, extending all around the new island, and possibly from a second unknown source close to 19.1789° S, 174.8348° W, 1.7 km E of Late’iki. Further periods of high hydrothermal activity are observed from 19 November to 24 December 2019 (corroborated by drone footage on 3 December, Fig. 2M), 8 January to 7 February, and 8 March to 17 April 2020 (Fig. 3). Maxar imagery shows elevated hydrothermal activity on 3 and 6 February, extending at least 6 km from the volcano and a very large hydrothermal plume extending more than 8 km on 7 March 2020 (Fig. 2l). All venting after 8 January appears to originate from 19.1786° S, 174.8505° W, within 60 m of the pre-eruption hydrothermal source, implying no significant relocation.

### Direct observations

#### Seafloor observations and sampling

Site inspection in February 2020 confirmed New Late’iki Island was submerged. Seafloor depths along a 740 m long AUV transect were all < 18 m, with the shallowest point of 8 m below sea level (19.1781° S, 174.8504° W, Fig. 1) corresponding to almost the midpoint between the pre- and post-2019 eruption islands. In cross-section, the summit morphology has a dome-shape (Fig. 1).

**Figure 4:**
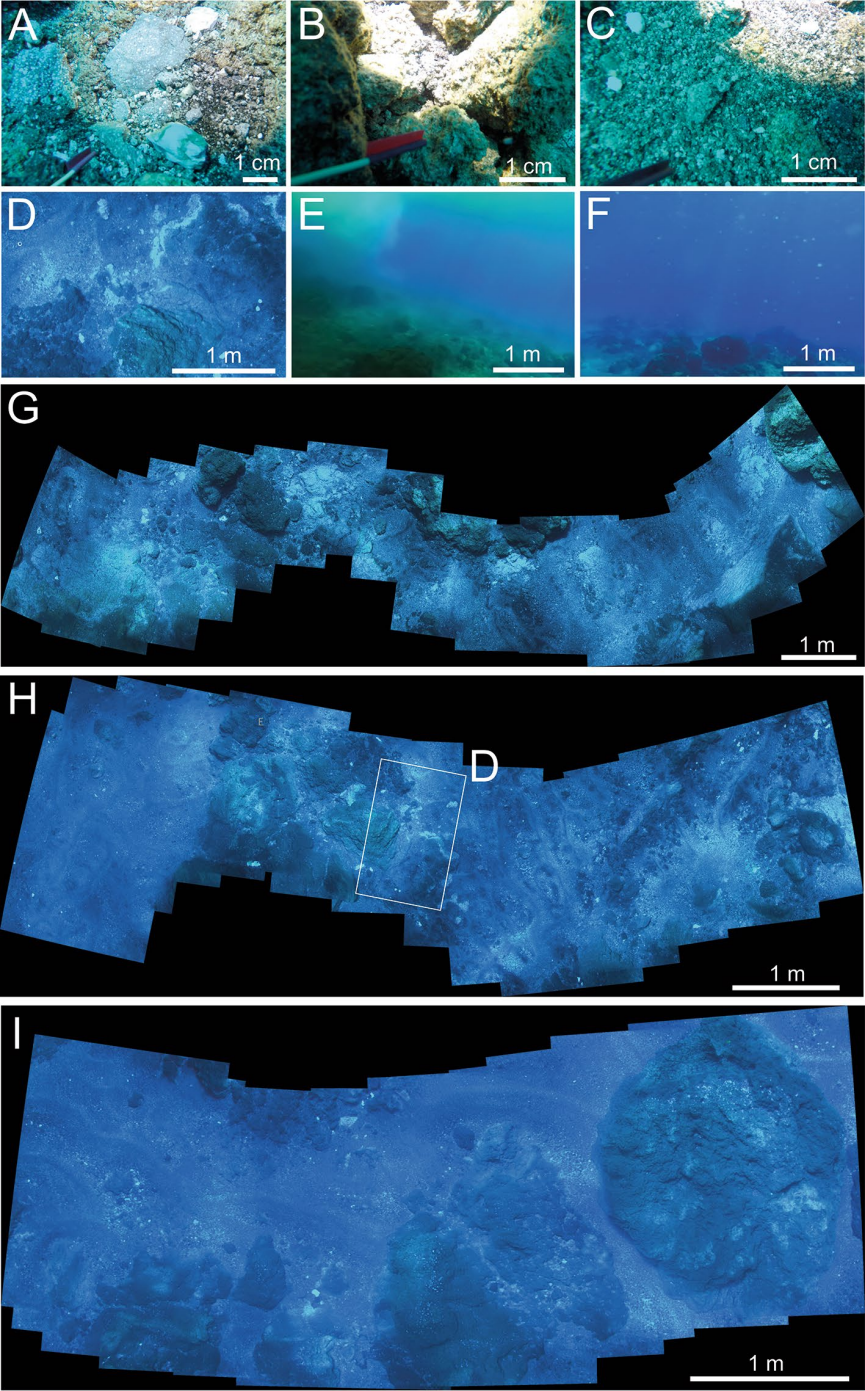
Seafloor imagery. (**A**–**C**) Images captured from sampling sites GS46 (**C**), GS48 (**B**) and GS49 (**A**) showing thin layers of fine-grained material and blocky lavas on the NE side of the summit. (**D**–**F**) Images taken by the UV along the track in Fig. 1 showing a range of summit features including altered cracks (**D**), hydrothermal vents (**E**) and bubbles in the water column (**F**). (**G**–**I**) Photographic mosaics of several images showing the nature of the seafloor across the summit, including in the area of New Late’iki Island.

The seafloor swath photographed with the AUV (Fig. 4) directly transects the area of October 2019 eruption activity (Fig. 1). A hydrothermal plume rising from the seafloor (Fig. 4E) caused reduced visibility; seafloor gas discharge (Fig. 4E,F) was imaged at several locations. In addition, the sampler-mounted camera imaged the sampling sites N and E of the AUV transect (Fig. 4A–C).

The underwater summit surface is characterised by 3 domains or lithofacies (Fig. 4). Facies 1 (Fig. 4D,G,H,I) comprises large intact masses of poorly vesicular lava^19^ with dimensions of several meters that exhibit planar flow banding in places. Lava surfaces only occasionally show a closely spaced prismatic to hackly jointing (Fig. 4I), but most are rough and irregular, standing up to 5 m above the surrounding seafloor. Irregular patches of white rock < 1 m across and discolouration around visible cracks/joints in the lava are interpreted as zones of alteration (Fig. 4D,G). Facies 2 (Fig. 4A,B) comprises scattered to clustered, rough-edged lava blocks ~ 30 cm to 1 m in size; only rarely do these poorly vesicular lava blocks exhibit curviplanar faces typically associated with quench fragmentation. Facies 3 (Fig. 4C) is a veneer of lapilli-sized, wave-reworked and mobilised, moderately sorted fragmental material where some lateral sorting is evident (finer grained at GS49 than at GS46, Fig. 1) that contained native sulphur. Drone footage of the last exposed surface of New Late’iki island on 3 December confirm the presence of Facies 2 (scattered lava blocks) and 3 (finely fragmented and wave-mobilised material) making up the remnants of New Late’iki (Fig. 2M).

Grab samples (Fig. 1) recovered ash- and lapilli-sized material that was a mixture of vesicular and glassy fragments and particles of native sulphur, typical of Facies 3 (Fig. 4C). One sample, a 10 cm cobble (GS49) (Fig. 4A) used for geochemical analysis, was a moderately vesicular glassy dacite lava, similar in appearance to larger blocks imaged on the seafloor, and texturally identical to the finer material sampled.

### 2019 Lava petrography and composition

**Figure 5:**
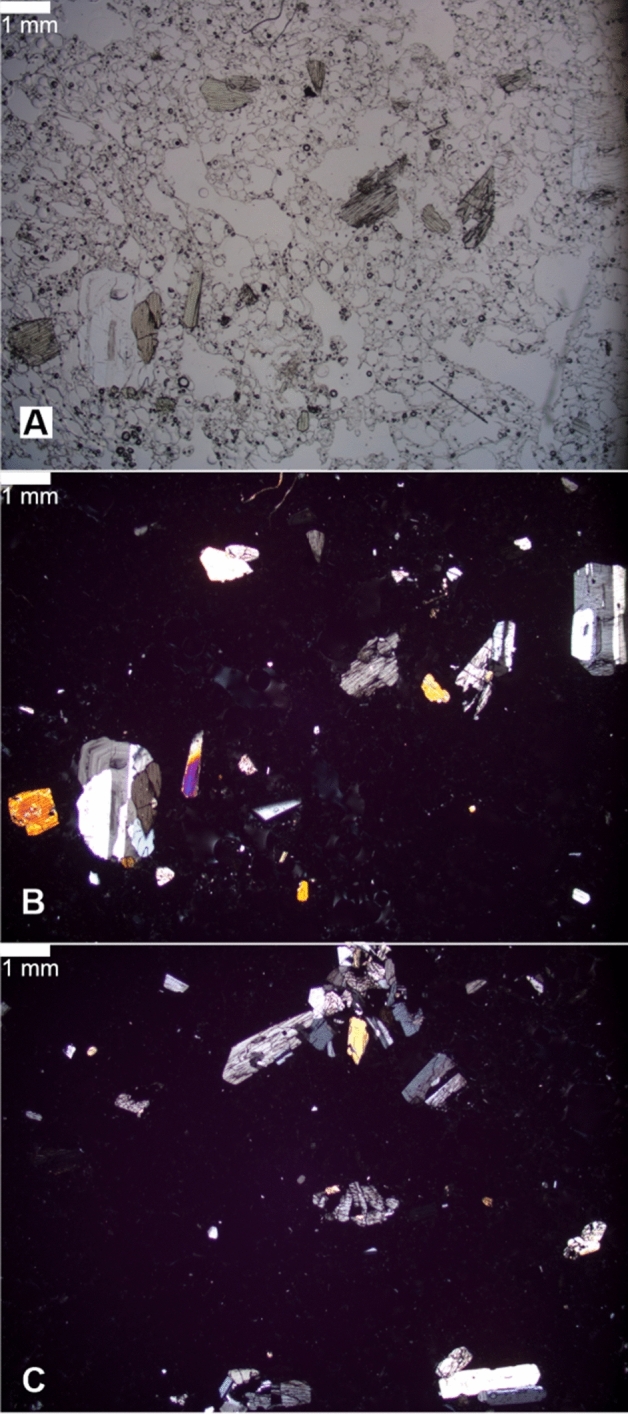
Photomicrographs of the 2019 Late’iki lava. (**A**) Plane polarised light view showing abundant, scattered pyroxene phenocrysts and highly vesicular glass. (**B**,**C**) Cross-polarised views showing polymineralic glomeroporphyritic textures, complexly zoned plagioclase and zoning in some pyroxene phenocrysts. Scale bar is 1 mm in all images.

Grab samples of the 2019 eruption deposits are clasts of grey lava that are finely vesicular, relatively crystal-rich with abundant glomerocrysts of plagioclase and pyroxene (Fig. 5), and are compared with lava from the 1967–8 eruption^2^. Crystallinity and phenocryst proportions are similar (26%, comprising 11.8% plagioclase, 6.5% clinopyroxene and 7.4% orthopyroxene^2^). Minor amounts of plagioclase microlites are present in the vesicular glass.

**Figure 6:**
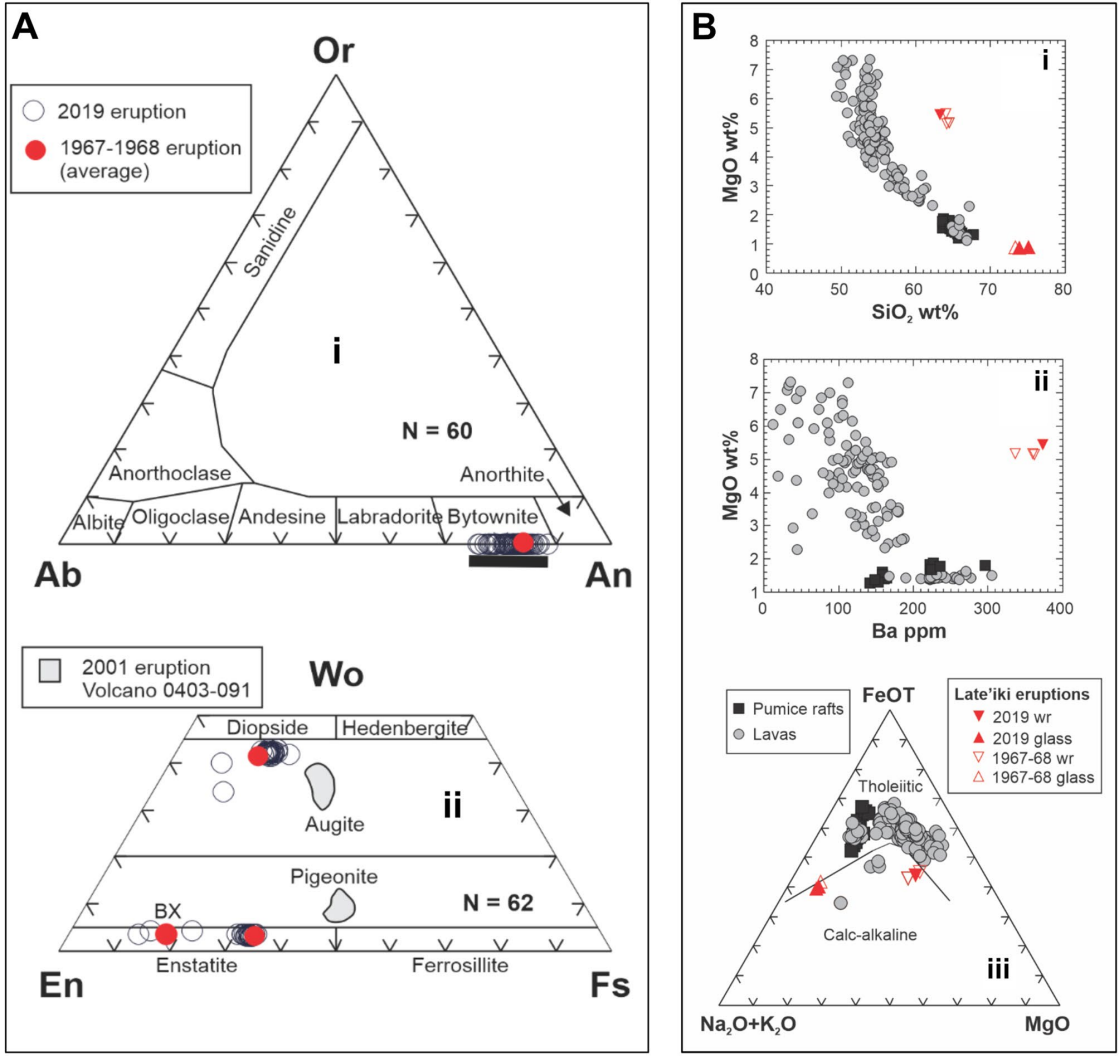
(**A**) Phenocryst compositions of the Late’iki lavas produced in the 1967–1968 and 2019 eruptions. Averaged compositions for the 1967–1968 lava are from Ewart et al.^2^. The data are further compared with phenocryst compositions from dacite pumice produced in the 2001 eruption of Volcano 0403-091, ~ 105 km to the north-northeast in the Tofua Arc^8^. (**i**) Feldspar phenocryst compositions. Range of plagioclase compositions from the 2001 eruption of Volcano 0403-091 indicated by black bar. *Ab* albite, *An* anorthite, *Or* orthoclase, *N* number of analyses. (**ii**) Pyroxene phenocryst compositions expressed in terms of the three-component system: wollastonite (Wo), enstatite (En), and ferrosillite (Fs), with field boundaries after Morimoto^61^. BX denotes the average composition of ‘bronzite’ xenocrysts identified by Ewart et al.^2^. Grey shade indicates the field of pyroxene compositions from the 2001 eruption of volcano 0403-091/Volcano F^8^. N is number of analyses. (**B**) Comparative geochemical plots of the Late’iki eruptions with erupted compositions from the Tofua arc (lavas are represented by grey filled circles, and pumice rafts-producing eruptions by blac-filled squares). (**I**) Harker plot of MgO vs SiO_2_ content showing the highly magnesian character of the 1967–2019 Late’iki dacite lavas, having an equivalent MgO content to basaltic andesites In the Tofua Arc. (**Ii**) MgO vs Ba plot illustrating the Late’iki lavas have distinctive trace element compositions and have the highest Ba contents of all analysed volcanics from the Tofua Arc. (**Iii**) AFM diagram comparing the Tofua Arc lavas and dacitic pumice compositions from pumice raft forming eruptions that form a typical tholeiitic trend. In contrast the Late’iki lavas exhibit chemical features resembling calc-alkali series rocks. Data for Tofua Arc lavas from^2–4^. Pumice raft compositions from^7,8,13,62^. Compositions of the 1967–1968 Late’iki lava from^2,15,26^.

The mineralogy of most glomerocrysts is consistent with a plagioclase-poor gabbronorite bulk composition. Plagioclase compositions are highly calcic showing a limited range from An_89-76_ (bytownite), similar to that of the 1967–8 lava (Fig. 6A), with little core to rim compositional variation despite evidence for complex oscillatory zoning (Fig. 5). Orthopyroxene occurs as both phenocrysts and in glomerocrysts (similar observations were made at Volcano F^8^ and Hunga Tonga-Hunga Ha’apai (Brenna et al., in press), with two distinct compositions (Fig. 6Aii): a dominant hypersthene (En_64_Fs_33_Wo_3_) and a rare Mg-rich bronzite (En_83_Fs_13_Wo_4_) that is occasionally rimmed by hypersthene and was interpreted by Ewart et al.^2^ as xenocrystic. Clinopyroxene typically occurs in glomerocrysts and exhibits two compositional populations: most is an unzoned calcic augite (En_41_Fs_17_Wo_42_) with the same composition as in the 1967–8 lava; we have also identified the minor occurrence of a phenocrystic Mg-rich augite (En_52_Fs_11_Wo_37_) that compositionally overlaps with augites in the basaltic andesite lavas^2^. Rare olivine is observed in the 2019 lava. Although not analysed in this study, rare, highly magnesian (Fo_93_) olivine phenocrysts with rims of hypersthene and Cr-spinel inclusions were reported in the 1967–8 lava^15^. We interpret the olivine, Mg-rich augite and bronzite to have to have been ultimately derived from basaltic magma and subsequently introduced into a very silicic melt.

Geochemically, Late’iki remains poorly characterised compared to other volcanoes in the Tofua arc (e.g.,^2,4,25^), with only a few analyses for the 1967–8 eruption^15,26,27^.

We show the 2019 lava is a low-K and low-Si dacite, similar to 1967–8 (Fig. 6B), and glass compositions are also rhyolitic as in 1967–8^15^; Table 1). Although dacitic, the Late’iki lavas are compositionally different to dacites erupted at Fonualei^2,3,8,13^. Tofua arc magmas define a characteristic Fe-enrichment or tholeiitic trend^2^ that includes the dacites except for those from Late’iki, which instead extend into the calc-alkaline field (Fig. 6B) due to much higher bulk rock MgO and CaO, and lower TiO_2_, Fe_2_O_3_T, and P_2_O_5_ contents. The comparison of the whole-rock versus glass compositions (Table 1) reveals that the anomalously high MgO, CaO and FeO contents result from the ferromagnesian crystal cargo.

**Table 1:** Summary of whole-rock and glass chemical analyses for the 1967–1968 and 2019 eruptions from Late’iki.

Name	NM11108	11108	MS11108	GS49	NM11108	GS49_1	GS49_2
Citation	Melson et al.^15^	Ewart et al.^2^	Ewart et al.^26^	This Study	Melson et al.^15^	This study	This study
Eruption	1967–1968	1967–1968	1968	2019	1967–1968	2019	2019
Sample	Lava	Lava	Lava	Lava	Glass	Glass	Glass
**Major elements (wt%)**							
SiO_2_	63.98	64.45	64.08	63.24	73.38	75.04	73.87
TiO_2_	0.32	0.39	0.39	0.39	0.45	0.51	0.49
Al_2_O_3_	12.94	12.57	12.50	12.72	12.66	11.95	12.98
Fe_2_O_3_	1.51	1.37	–	7.51	1.11	–	–
FeO	5.43	5.28	–	–	2.84	3.61	3.51
Fe_2_O_3_T	7.54	7.24	7.20	7.51	–	4.01	3.90
MnO	–	0.12	0.12	0.13	0.06	0.06	0.05
MgO	5.47	5.16	5.14	5.45	0.84	0.83	0.80
CaO	7.51	7.06	7.01	7.38	3.79	3.43	4.08
Na_2_O	1.83	2.61	2.59	2.18	3.09	2.99	3.09
K_2_O	0.95	0.91	0.91	0.93	1.52	1.61	1.52
P_2_O_5_	0.06	0.07	0.07	0.07	0	0.07	0.05
Cl	–	–	–	–	–	0.10	0.10
LOI	0	0.91	0.91	0.32	0.16	–	–
**Trace elements (ppm)**							
Ba	334	360	362	373	–	–	–
Rb	–	14	14	16	–	–	–
Sr	–	140	140	138	–	–	–
Y	–	21	21	18	–	–	–
Zr	–	46	46	39	–	–	–
Nb	5	–	0	3	–	–	–
Th	–	–	0	1	–	–	–
Pb	–	–	4	4	–	–	–
Ga	–	14	11	–	–	–	–
Zn	–	58	66	58	–	–	–
Cu	–	88	88	127	–	–	–
Ni	–	53	53	41	–	–	–
V	–	175	175	179	–	–	–
Cr	–	230	249	166	–	–	–
Hf	–	–	1	–	–	–	–
Cs	–	–	1	–	–	–	–
Sc	–	29	31	–	–	–	–
Ta	–	–	0	–	–	–	–
Co	–	25	28	24	–	–	–
U	–	–	0	–	–	–	–
La	3	–	3	–	–	–	–
Ce	7	–	7	8	–	–	–
Pr	–	–	1	–	–	–	–
Nd	–	–	5	–	–	–	–
Sm	2	–	1	–	–	–	–
Eu	0	–	0	–	–	–	–
Gd	2	–	2	–	–	–	–
Tb	–	–	0	–	–	–	–
Dy	3	–	3	–	–	–	–
Ho	–	–	1	–	–	–	–
Er	2	–	2	–	–	–	–
Tm	–	–	–	–	–	–	–
Yb	2	–	2	–	–	–	–
Lu	–	–	0	–	–	–	–

Whole-rock data normalised on an LOI-free basis. Data for the 1967–1968 eruption from Melson et al.^15^ and Ewart et al.^2,27^.

**Figure 7:**
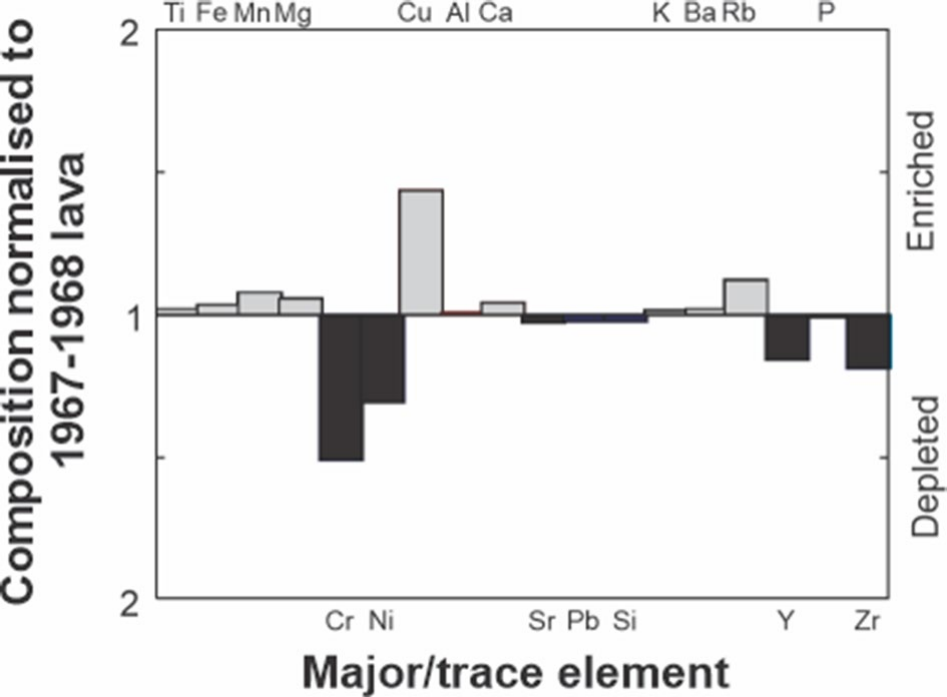
Enrichment-depletion diagram showing the variation in major and trace elements for eruptions at Late’iki. The composition of the 2019 eruption is normalised to the composition of the 1967–1968 eruption^26^. The diagram reveals only subtle differences in a few elements between 2019 and the 1967–1968 eruption, most of which is within analytical error.

Comparison of the 1967–8 and 2019 lava compositions enables us to assess potential changes in the Late’iki magma system over the last 50 years (Table 1). An enrichment-depletion diagram (Fig. 7) shows the 2019 lava is almost identical within analytical uncertainties (Table 1); trace element abundances are also similar but Cr is distinctly lower (Fig. 7). Elevated Cr abundances in dacitic magmas can indicate the presence of basaltic magma mixing^28^, previously recognised at Late’iki by the presence of xenocrystic olivine^2,15^. However, Cr abundances are slightly lower in the 2019 lava (Fig. 7). The lack of depletion in compatible elements such as CaO, Sr, MgO or enrichment in incompatible elements including SiO_2_ argue against any fractionation of the magma since 1967–8. Thus, the available geochemical data indicate the same magma that began erupting in 1967 continues to erupt, feeds the hydrothermal system at Late’iki and remains largely unmodified over the last 50 years.

FTIR analysis of the 2019 matrix glass reveals it has an H_2_O content of 0.11 wt%. All water exists as the hydroxyl species (OH); no molecular water (H_2_O_m_) was detected. H_2_O content and speciation in magma vary with pressure and temperature^29–32^. These data are consistent with the subaerial island-forming eruption and vesicular character of the lava where magma has degassed to atmospheric pressure prior to eruption and quench^32,33^. The lack of H_2_O_m_ also confirms the sample was unaffected by post-eruption secondary hydration (e.g.^34,35^) from surrounding seawater or hydrothermal fluids, consistent with the slow diffusivity of H_2_O in glass at low temperatures (e.g.^36,37^) and the short timescale between eruption and sample collection (~ 4 months).

## Discussion

### Precursory activity

Like many marine volcanoes, Late’iki is poorly covered by regional seismic stations and no seismic events were recorded prior to the eruption^17^. The only time series data that can be reliably derived from satellite observations prior to the eruption are the size and shape of the island, and the extent of hydrothermal venting from the summit (Fig. 3). No meaningful changes in island area are observed but increases in hydrothermal outflow occur leading up to the eruption (Fig. 3). Hydrothermal activity clearly increased before the eruption, however, this is not unique as the previous years had three or four increases in hydrothermal discharge intensity, in each case starting at low levels and becoming progressively more vigorous for a period of 5–8 months before dropping back to low levels (Fig. 3). High intensity hydrothermal venting was observed over several weeks in August 2019 and outflow right before the 2019 eruption was slightly higher than in previous periods of activity, but this increase in hydrothermal flux would be hard to distinguish from earlier increases in flux that were not followed by an (observed) eruption without other supporting information. The increases in hydrothermal outflow could be tied to magma chamber recharge, but based on geochemical data most likely reflect the continued rise and degassing of magma that first entered the plumbing system in 1967.

### The October 2019 eruption

Establishing Volcanic Explosivity Indices (VEIs)^38^ for submarine eruptions is challenging because it is difficult to estimate volumes of erupted material deposited underwater and they cannot be directly applied to effusive eruptions, which would typically be a VEI of 0–1. Estimating the volume of material accumulated in the 2019 eruption is also challenging but based on the pre-existing bathymetry is likely to be ~ 10^3^ m^3^. This low-explosivity, effusive eruption style contrasts with surrounding volcanoes, e.g. Home Reef 2006 (VEI 2), Volcano-F 2019 (VEI 2–3), Late 1854 (VEI 2), and Kao 1847 (VEI 2).

Although described as ‘Surtseyan’^16^, satellite images do not record the characteristic dense black ‘cock’s comb’ tephra jets associated with that style of emergent, typically basaltic, volcanism^39^. Instead, the 2019 eruption mostly generated only tephra-poor, steam plumes (Fig. 2C,D). Such steam plumes are consistent with our interpretation of the fragmented material from the 2019 eruption as being driven by phreatic explosions, while dome forming eruptions are commonly low-explosivity and steam and ash-poor. Passive steam release as a result of heating of ocean water from contact with extruding lava will also have been persistent throughout the eruption.

As evidenced by satellite imagery and bubbles observed in post-eruption seafloor photography (Fig. 4), Late’iki is a hydrothermally active volcano (Fig. 2) and summit venting makes this outflow more apparent than other volcanoes in the region. The same duration or intensity of hydrothermal venting was not observed preceding or following the 2019 eruption of Volcano 0403–019 (also known as Volcano F)^9^ although venting did continue for several years after the shallow Home Reef eruption. Persistently degassing subaerial volcanoes are often characterised by mildly explosive eruptions because outgassing during periods of quiescence reduces pressures within magma plumbing systems by several MPa over relatively short (month–year) time periods, and increases magma viscosities, which may stall a rising magma batch^40^. However, if not stalled, in a situation where exsolved gasses have not fully escaped the conduit system, higher viscosities can enhance explosivity^41,42^, and mineralisation and hydrothermal sealing of volcanic rocks^43^, particularly within lava domes^44,45^, may allow greater pressurisation of the system and more explosive eruptions. Thus, while the hydrothermal system at Late’iki may currently contribute to the lower explosivity of its eruptions compared to the similarly shallow dacitic Home Reef and 0403-091 volcanoes, the hydrothermal system here has the potential to enhance explosivity under other conditions.

The overlap in phenocryst mineralogy and composition, glass and bulk-rock chemistry indicate the 2019 eruption has continued to evacuate magma initially injected into the volcanic system as recorded by the 1967–1968 eruption, with no significant fractionation occurring over the last 50 years. Since 1967, eruptions at Late’iki have been relatively closely spaced, occurring at intervals of 11, 11, 5 and 24 years. A similar cluster of eruptions occurred at Late’iki from 1851 to 1886, and the more substantial hiatus from 1886 to 1967 suggests the 1967 eruption recorded the eruption of a new magma batch. The presence of a minor ultramafic mineral assemblage, headlined by forsteritic olivine in a rhyolite glass, suggests mafic recharge may have been responsible for eruption initiation in 1967–68. The bulk rock chemical characteristics of the Late’iki lavas are highly unusual and apparently unique in the Tofua arc (Fig. 7). Although the rhyolitic glass composition is consistent with the general trend and a liquid line of descent for Tofua arc magmas, the bulk-rock composition has been significantly modified by the crystal cargo attesting to open system and magma recharge at Late’iki. While the Late’iki lavas are crystal-rich and dominated by glomerocrystic textures, two traits commonly used to interpret mush-melt extraction^46,47^, the highly magnesian mineral assemblage is strongly out of equilibrium with the rhyolite glass and argues against simple melt extraction from a crystal mush as represented by the glomerocrysts.

### Eruption style and volcanic products of the 2019 eruption

The new unrest of Late’iki in October 2019 resulted from renewed lava extrusion. However, because of the shallow water setting of the volcano, eruption style was not simply effusive and explosive water interaction also occurred. Fragmentation to produce the lava blocks and finer ash- and lapilli-sized particles is interpreted to result primarily from phreatic steam-driven explosivity due to hot lava-water interaction. Scarcity of curviplanar-faced lava fragments suggests that non-explosive quench fragmentation processes^48^ associated with hyaloclastite formation did not dominate. Further subsequent breakage and abrasion from wave action is also likely, as observed for similarly pumiceous lavas from the 1967–1968 eruption^15^. The interpretation of explosive phreatic activity is consistent with observations of both the 1967–8 eruption, where steam plumes were associated with explosions resulting in ballistic fallout of lava blocks^15^, and the 1995 eruption, where ash-laden steam plumes extended up to 500 m height and phreatic explosions occurred during collapse of the active lava flow front^17^.

The observed lithofacies at the summit of Late’iki contrast with current facies models for silicic volcanoes transiting from being submerged to subaerial^49^. Submarine to emergent dacitic lava domes are observed to consist of a massive coherent core, a flow-banded outer zone, an in situ brecciated margin and an enveloping carapace of hyaloclastite produced by quench fragmentation of the lava dome margins^49–51^. Although we observe intact massive to flow banded lava, the abundant monomict lava fragment breccias are mostly absent and four months after eruption only a thin veneer of wave-reworked, finely fragmented material directly overlying submerged coherent lava remains. Drone observations on 3 December, ~ 7 weeks after the eruption (Fig. 2M), showed a more significant volume of finely fragmented material. This suggests wave action has efficiently mobilised substantial volumes of finely fragmented lava material to deeper water. Any hyaloclastite debris may have also been remobilised and transported into deeper water beyond our survey area, but we consider fragmentation processes in this emergent environment to have instead been dominated by steam-driven explosivity (i.e. phreatic explosions). Similar observations of little to no loose material on and around the volcano summit were also made following the 1967–8 eruption^15^. This has implications for interpreting the paleoenvironment for very shallow marine to emergent lava flows and domes where diagnostic textural evidence of water-magma interaction (i.e. hyaloclastite) may not be developed in sufficient quantities, and finely fragmented material from phreatic explosions is quickly remobilised and removed from the eruption site.

### Activity post-October 2019 and the destruction of New Late’iki

On at least three dates after the main eruptive phase atypical activity is imaged at Late’iki by satellites. It is possible that other processes could explain several of these phenomena observed after the main eruptive phase. The concentric waves could be produced by refraction around a shallow region of the volcano, but because they are not present in other images and do not correlate with periods of anomalous wind or wave height this would require a shallowing and then deepening of the seafloor in this region on a short timescale (days to weeks) which would itself indicate active volcanic extrusion. Similarly, the white water could be produced by wave breaks on shallow seafloor but again these observations do not correlate with anomalous weather conditions and white water is not present in images taken within a few days of the inferred events. We therefore interpret the white water and concentric rings as evidence of intermittent, weak explosive activity continuing after October 2019, most likely resulting from steam explosions caused by seawater infiltration. The presence of pumiceous lava stringers in January may suggest a minor amount of newly fragmented material at this time but it is not definitive, since floating material could potentially be produced from the vesicular lava carapace at shallow water depths either by passive buoyant detachment from an active flow (e.g.^52^) or explosive phreatic disruption of previously emplaced lavas. In any case, some ongoing phreatic or volcanic activity is required to explain these observations into 2020.

A rapid reduction in size of New Late’iki Island is observed between 30 October and 19 November^17^, followed by much slower degradation until its disappearance on 14 December (Fig. 2). While the rapid rates of erosional destruction of the New Late’iki Island reflect wave remobilisation of unconsolidated material, direct observation and sampling confirm the unconsolidated material is not strictly of pyroclastic origin as previously interpreted^17^ but comprises finely fragmented lava produced by steam-driven explosivity. Satellite imagery on 19 November and 3 January records continued but intermittent phreatic explosions that continued to modify the summit of Late’iki.

## Conclusions

By combining direct observation and sampling of the post-eruptive summit with satellite observations, we are better able to understand the 2019 Late’iki eruption, highlighting the need for in-situ observations and sampling. The only observable indication that Late’iki was about to erupt was an 8-month non-unique increase in hydrothermal discharge. The eruption that destroyed the pre-existing island and formed the ephemeral New Late’iki was characterised by tephra-poor steam plumes. Although the main eruptive phase ended by 23 October 2019, episodic, minor explosive activity continued into 2020. Crucially, our direct post-eruption observations and sampling of the seafloor enable us to ground truth interpretations of satellite imagery and provide new insights into eruption mechanisms. The summit in February 2020 comprised large masses of intact, vesicular to locally flow-banded dacitic lava blanketed in a thin veneer of finely fragmented lava debris. We thus interpret the 2019 eruption as being predominantly effusive, with relatively minor explosive activity driven by phreatic, i.e. steam-driven fragmentation of extruded lava. The low explosivity of this eruption is interesting and highlights the need for a better understanding of hydrothermal modulation of explosivity both here and elsewhere. We find little evidence for hyaloclastite formation by quench fragmentation of the lava, suggesting it was a minor process or that resulting deposits are swiftly removed from the summit in shallow to emergent settings. Further evidence from geochemical analysis of retrieved samples reveals matrix glass volatile contents consistent with degassing to atmospheric pressure during subaerial emplacement and shows little change in magma composition since the 1967–8 eruption. This suggests the same magma batch is continuing to move through the Late’iki plumbing system. The 15 January 2022 explosive eruption at Hunga Tonga–Hunga Ha’apai demonstrated the potential for powerful and regionally hazardous eruptions in the Tofua arc and highlights the need for active monitoring alongside a more comprehensive understanding of the volcanic history and magma plumbing systems in this very active region.

## Methods

### Satellite imagery

Data from three different satellite observation programs were used to reconstruct the timings and phases of the 2019 eruption. The European Space Agency’s Sentinel 2 Satellites’ Level 1C products (10 m per pixel) is pre-processed into 100 km^2^ tiles projected into cartographic coordinates using a digital elevation model. More frequent but lower resolution data were provided by the NASA Worldview portal, which collates Earth observation data from NASA’s constellation of Earth observing satellites. The eruption was visible in data collected using the MODIS instrument operating on the Terra spacecraft, which measures 36 spectral bands and acquires data at 250 m, 500 m, and 1000 m spatial resolutions. Geolocation for this data is performed by the MODIS Adaptive Processing System (MODAPS) with an accuracy of around 20 m. Additional sporadic, but very high resolution (as good as 30 cm per pixel) imagery was provided by the Maxar technologies DigitalGlobe platform collected using their WorldView-2 and WorldView-3 satellites^53^. Data were projected and compared using the open-source software QGIS (v. 3.0.3 Girona; QGIS Development Team^54^). QGIS was also used to define island locations and boundaries and to measure the subaerial area of the 1995 island before the 2019 eruption and of New Late’iki for the period after the 2019 eruption until it because completely submerged.

### Hydrothermal activity

We use the satellite imagery to qualitatively identify four different levels of hydrothermal activity. These are: No Activity—no discoloured water in the vicinity of the volcano (e.g. Fig. 2A); Low Activity–small amounts of discoloured water visible directly proximal to the volcano, very little flow away from the source/around the edifice (not shown in Fig. 2); Intermediate Activity—clearly visible areas of discoloured water extending away from the source/around the edifice (e.g. Figure 2!); and High Activity—extensive flow away from the source/around the edifice forming large areas of discoloured water that, where they interact with currents, remain visible at substantial distances (several km) from the edifice (e.g. Fig. 2L).

### Data sources

SO_2_: National Aeronautics and Space Administration Global Sulfur Dioxide Monitoring Home Page https://so2.gsfc.nasa.gov/index.html

Hysplit https://www.ready.noaa.gov/HYSPLIT.php

GMRT https://www.gmrt.org/

DigiGlobe/Maxar https://discover.digitalglobe.com/

### 27 February 2020 Late’iki summit survey

Seafloor imagery was collected using the autonomous underwater vehicle RangerBoT^55^ owned and operated by the Queensland University of Technology. Originally designed for shallow water reef surveys, the robot was repurposed to study the shallow summit of the volcano. Both downward and forward-facing cameras captured imagery from an altitude of between 3 and 5 m above the seafloor on pre-programmed mission course running over the shallowest area of the summit mapped from the satellite data. RangerBoT was deployed and recovered from a small sports fishing boat and navigation and obstacle avoidance was provided by real-time on-board vision calculations.

Sampling was conducted of the seafloor using a small grab sampler owned and operated by the Department of Geology and Palaeontology, National Museum of Nature and Science, Japan. Working conditions were challenging, with no accurate post-eruptive bathymetry available and changes in currents and tides causing the hydrothermal plume to move around the volcano during the day. As a result, sample sites were chosen opportunistically to the north west of the volcano, along the path of approach to allow for easy evacuation in the event of any heightened activity. Most sampling sites were NE of the former Late’iki and New Late’iki islands yet still within the area of eruptive activity imaged on 20 October (Fig. 1). A 4 k resolution Go-Pro camera was mounted on the sediment grab to provide ground truthing data of the seafloor. Drone footage of the almost submerged island was collected on the 3^rd^ December (by Darren Rice of Matafonua Lodge) and the full videos can be found at https://figshare.com/s/a076ae26e952dafa51be.

Geochemical data were collected on the one recovered summit sample large enough for comprehensive analysis, using XRF and EPMA (Appendix X.3) at The National Museum of Nature and Science and FTIR at JAMSTEC. Sample preparation and analytical procedures for XRF follow those described in Sano et al.^56^. The EPMA (JEOL JXA-8230) analysis was conducted using an accelerating voltage of 15 kV, a probe current of 12 nA, and a spot diameter of 1 μm for mineral analysis. For glass analysis, accelerating voltage of 15 kV, a probe current of 8 nA, and a spot diameter of 10 μm was used to minimize Na loss. FTIR analysis was conducted on individual crushed shards as in Mitchell et al.^57^, using the mid-IR 3500 cm^−1^ H_2_O_t_ and 1630 cm^−1^ H_2_O_m_ absorbance peaks with a species-dependent molar absorptivity coefficient for the 3500 cm^−1^ peak^35^.

## Supplementary Information

Supplementary Information 1.

Supplementary Information 2.
