# Supplementary material for: The 2019–2020 volcanic eruption of Late’iki (Metis Shoal), Tonga
Source: Sci Rep. 2022 May 6;12:7468. doi: 10.1038/s41598-022-11133-8 (PMC9076857; doi:10.1038/s41598-022-11133-8)
Supplement: Supplementary file 1 — Supplementary Information 1. [file 41598_2022_11133_MOESM1_ESM.pdf]

The 2019-20 volcanic eruption of Late'iki (Metis Shoal), Tonga

I. A. Yeo[1*], I. M. McIntosh[2], S. E. Bryan[3], K. Tani[4], M. Dunbabin[5], D. Metz[2], P. C. Collins[6], K. Stone[7] and M. S. Manu[8]

[1] National Oceanography Centre Southampton, UK, i.yeo@noc.ac.uk

[2] Japan Agency for Marine-Earth Science and Technology (JAMSTEC), Japan

[3] School of Earth & Atmospheric Sciences, Queensland University of Technology, Australia

[4] National Museum of Nature and Science (Kahaku), Japan

[5] School of Electrical Engineering & Robotics, Queensland University of Technology, Australia

[6] Queens University Belfast, UK

[7] Vava'u Environmental Protection Association (VEPA), Tonga

[8] Natural Resources Division, Ministry of Lands and Natural Resources, Tonga

Appendix X.X
Summary of observations of the 2019-2020 volcanic eruption of Late'iki made by various sources.

DATE	SOURCE	OBSERVATIONS
**10/10/2019**	ESA Sentinel-2	1995 Island present, no volcanic activity, vigorous hydrothermal flow to the west
	NASA MODIS	No evidence of volcanic activity, plume not visible in this lower resolution data
**11/10/2019**	NASA MODIS	No evidence of volcanic activity, plume not visible in this lower resolution data
**12/10/2019**	NASA MODIS	Steam plume visible extending W from volcano, discolouration in surrounding water
**13/10/2019**	NASA MODIS	Smaller steam plume extending W from vocano, discolouration in surrounding water
**14/10/2019**	NASA MODIS	Steam plume visible extending W from volcano, discolouration in surrounding water
	Vessel	Eruption in progress
**15/10/2019**	ESA Sentinel-2	Volcanic eruption in progress, island larger but mostly obscured by steam plume, seawater discolouration surrounding the volcano, thermal signature
	NASA MODIS	Strong steam plume extending SW from volcano, discolouration in surrounding water
	Real Tonga Flight	Large steam plume rising from the eruption
**19/10/2019**	NASA MODIS	Strong steam plume extending W from volcano, discolouration in surrounding water
**20/10/2019**	ESA Sentinel-2	Volcanic eruption in progress, island entirely obscured by steam plume, seawater discolouration surrounding the volcano, thermal signature
	NASA MODIS	Steam plume extending NW from volcano, discolouration in surrounding water
**21/10/2019**	NASA MODIS	Strong steam plume extending NW from volcano, discolouration in surrounding water
**22/10/2019**	NASA MODIS	Steam plume extending W from volcano, discolouration in surrounding water
**25/10/2019**	ESA Sentinel-2	No evidence of extrusion, island mostly obscured by cloud, vigorous hydrothermal flow to NW
**30/10/2019**	ESA Sentinel-2	New Late'iki Island unobscured, hydrothermal flow to the NE
	NASA MODIS	Small amount of discolouration in surrounding water
**06/11/2019**	NASA MODIS	Discolouration in water and plume to the W
**07/11/2019**	NASA MODIS	Discolouration in water surrounding the volcano
**08/11/2019**	NASA MODIS	Small amount of discolouration in surrounding water

**09/11/2019**	ESA Sentinel-2	New Late'iki island reduced in size but still visible, vigorous hydrothermal flow around the island
	NASA MODIS	Fairly extensive discolouration in water surrounding the volcano
**13/11/2019**	NASA MODIS	Small amount of discolouration in surrounding water
**14/11/2019**	ESA Sentinel-2	New Late'iki island reduced in size but still visible, vigorous hydrothermal flow around the island and to the S
	NASA MODIS	Discolouration in water and plume to the S
**15/11/2019**	NASA MODIS	Small amount of discolouration in surrounding water
**19/11/2019**	ESA Sentinel-2	New Late'iki island still visible, white water visible 260 m south of the island forming the centre of concentric ring waves, vigorous hydrothermal venting to the south
	NASA MODIS	Discolouration in water and plume to the W
**20/11/2019**	NASA MODIS	Small amount of discolouration in surrounding water
**24/11/2019**	ESA Sentinel-2	New Late'iki Island still visible, no other white water, vigorous hydrothermal venting to the SE
**26/11/2019**	NASA MODIS	Small amount of discolouration in surrounding water
**27/11/2019**	NASA MODIS	Small amount of discolouration in surrounding water
**28/11/2019**	NASA MODIS	Small amount of discolouration in surrounding water
**29/11/2019**	NASA MODIS	More extensive discolouration in surrounding water and plume to the E
**03/12/2019**	Drone footage	No volcanic activity, New Late'iki Island reduced to sea level covered by lapilli to ash size fragmented lava and large metre sized blocks of dacite
**06/12/2019**	NASA MODIS	Small amount of discolouration in surrounding water
**07/12/2019**	NASA MODIS	No obvious discolouration in surrounding water
**08/12/2019**	NASA MODIS	No obvious discolouration in surrounding water
**09/12/2019**	ESA Sentinel-2	New Late'iki Island still visible, no other white water, vigorous hydrothermla venting
**10/12/2019**	NASA MODIS	Small amount of discolouration in surrounding water
**13/12/2019**	NASA MODIS	Small amount of discolouration in surrounding water
**14/12/2019**	ESA Sentinel-2	New Late'iki island mostly submerged, some wave breaks in previous location, vigorous hydrothermal venting to the south
**20/12/2019**	NASA MODIS	No obvious discolouration in surrounding water
**21/12/2019**	NASA MODIS	No obvious discolouration in surrounding water
**23/12/2019**	NASA MODIS	No obvious discolouration in surrounding water
**24/12/2019**	ESA Sentinel-2	New Late'iki island mostly submerged, some wave breaks in previous location, vigorous hydrothermal venting to the north
**02/01/2020**	NASA MODIS	Small amount of discolouration in surrounding water

**03/01/2020**	ESA Sentinel-2	New Late'iki Island now totally submerged, white water and concerntric waves visible slightly to the south of the previous island location, possible steam plume
	MAXAR Worldview-2/3	White water, possible steam plume, discoloured water forming two plumes to the SW, ripples visible to th E of the white water
	NASA MODIS	Possible small steam plume and discolouration in water forming plume to W
**06/01/2020**	NASA MODIS	Moderate discolouration in surrounding water
**08/01/2020**	ESA Sentinel-2	New Late'iki island completely submerged, vigorous hydrothermal venting to the N and S
	NASA MODIS	Moderate discolouration in surrounding water
**09/01/2020**	NASA MODIS	Small amount of discolouration in surrounding water
**10/01/2020**	NASA MODIS	Moderate discolouration in surrounding water
**15/01/2020**	NASA MODIS	Small amount of discolouration in surrounding water
**21/01/2020**	NASA MODIS	No obvious discolouration in surrounding water
**22/01/2020**	MAXAR Worldview-2/3	Vigorous venting forminga plume to the E, stringers of material floating within a few km of the volcano
	NASA MODIS	No obvious discolouration in surrounding water
**23/01/2020**	ESA Sentinel-2	New Late'iki island completely submerged, vigorous hydrothermal venting to N and S
	NASA MODIS	Small amount of discolouration in surrounding water
**28/01/2020**	ESA Sentinel-2	New Late'iki island completely submerged, vigorous hydrothermal venting to N and S
**01/02/2020**	NASA MODIS	No obvious discolouration in surrounding water
**02/02/2020**	ESA Sentinel-2	New Late'iki island completely submerged, vigorous hydrothermal venting to S
	NASA MODIS	No obvious discolouration in surrounding water
**03/02/2020**	NASA MODIS	Small amount of discolouration in surrounding water
**06/02/2020**	NASA MODIS	Small amount of discolouration in surrounding water
**07/02/2020**	ESA Sentinel-2	New Late'iki island completely submerged, vigorous hydrothermal venting to the NW
**07/03/2020**	MAXAR Worldview-2/3	Very vigorous hydrothermal venting forming a plume to the NW

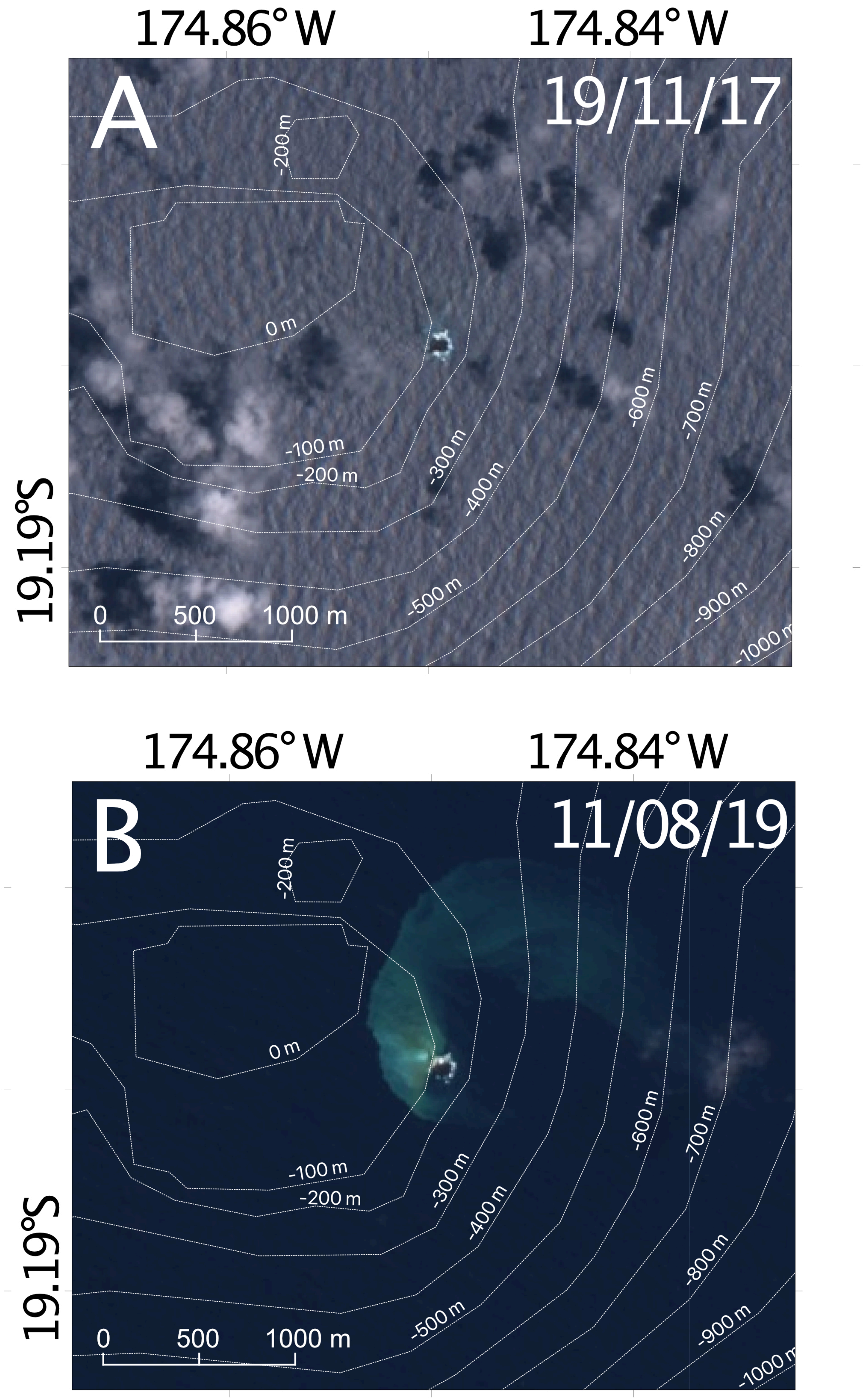

174.86° W
174.84° W
A
19/11/17
-200 m
0 m
-100 m
-200 m
-300 m
-400 m
-500 m
-600 m
-700 m
-800 m
-900 m
-1000 m
19.19°S
0
500
1000 m
174.86° W
174.84° W
B
11/08/19
-200 m
0 m
-100 m
-200 m
-300 m
-400 m
-500 m
-600 m
-700 m
-800 m
-900 m
-1000 m
19.19°S
0
500
1000 m

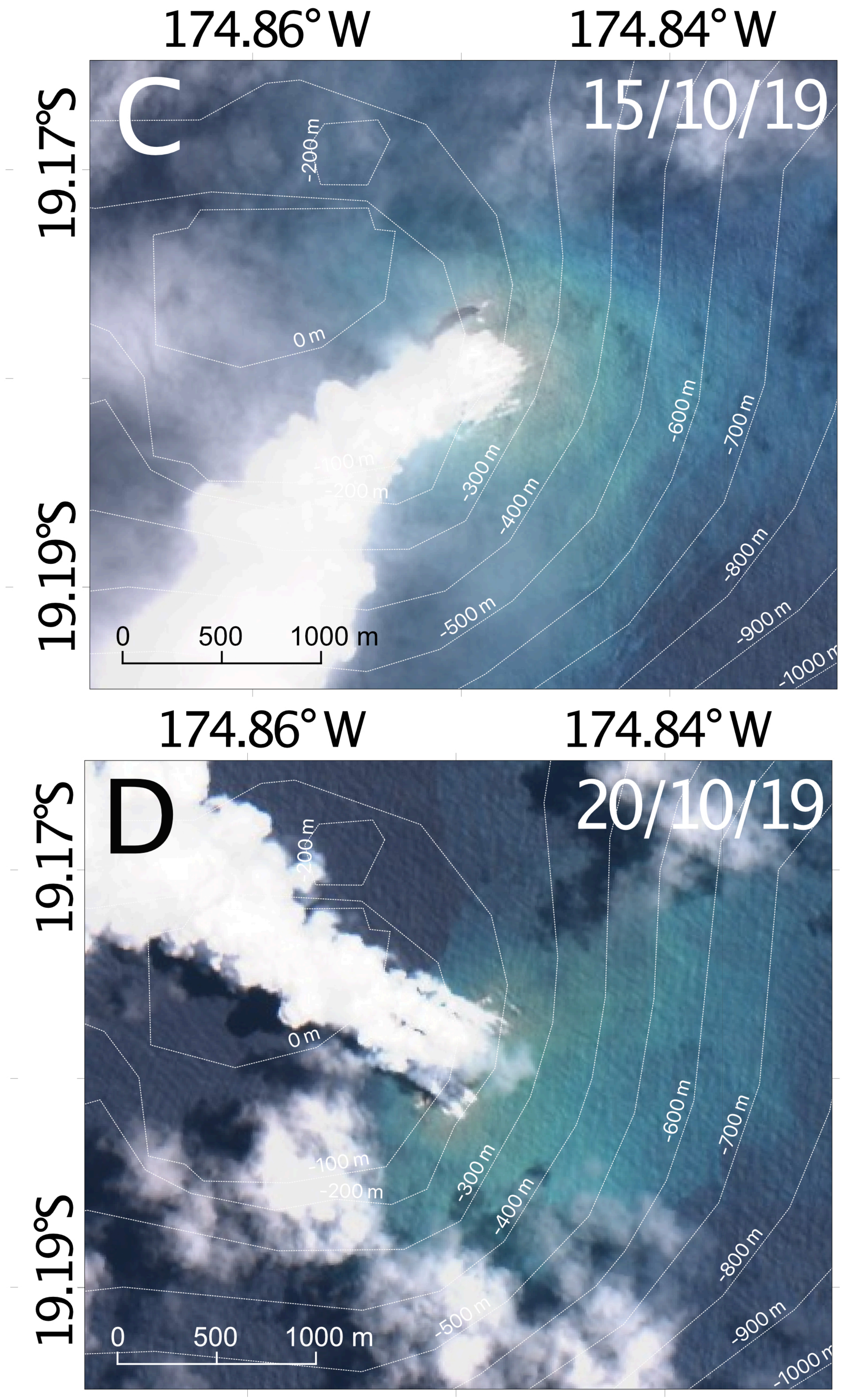
174.86° W
174.84° W
C
15/10/19
19.17°S
19.19°S
-200 m
0 m
-100 m
-200 m
-300 m
-400 m
-500 m
-600 m
-700 m
-800 m
-900 m
-1000 m
0
500
1000 m
174.86° W
174.84° W
D
20/10/19
19.17°S
19.19°S
-200 m
0 m
-100 m
-200 m
-300 m
-400 m
-500 m
-600 m
-700 m
-800 m
-900 m
-1000 m
0
500
1000 m

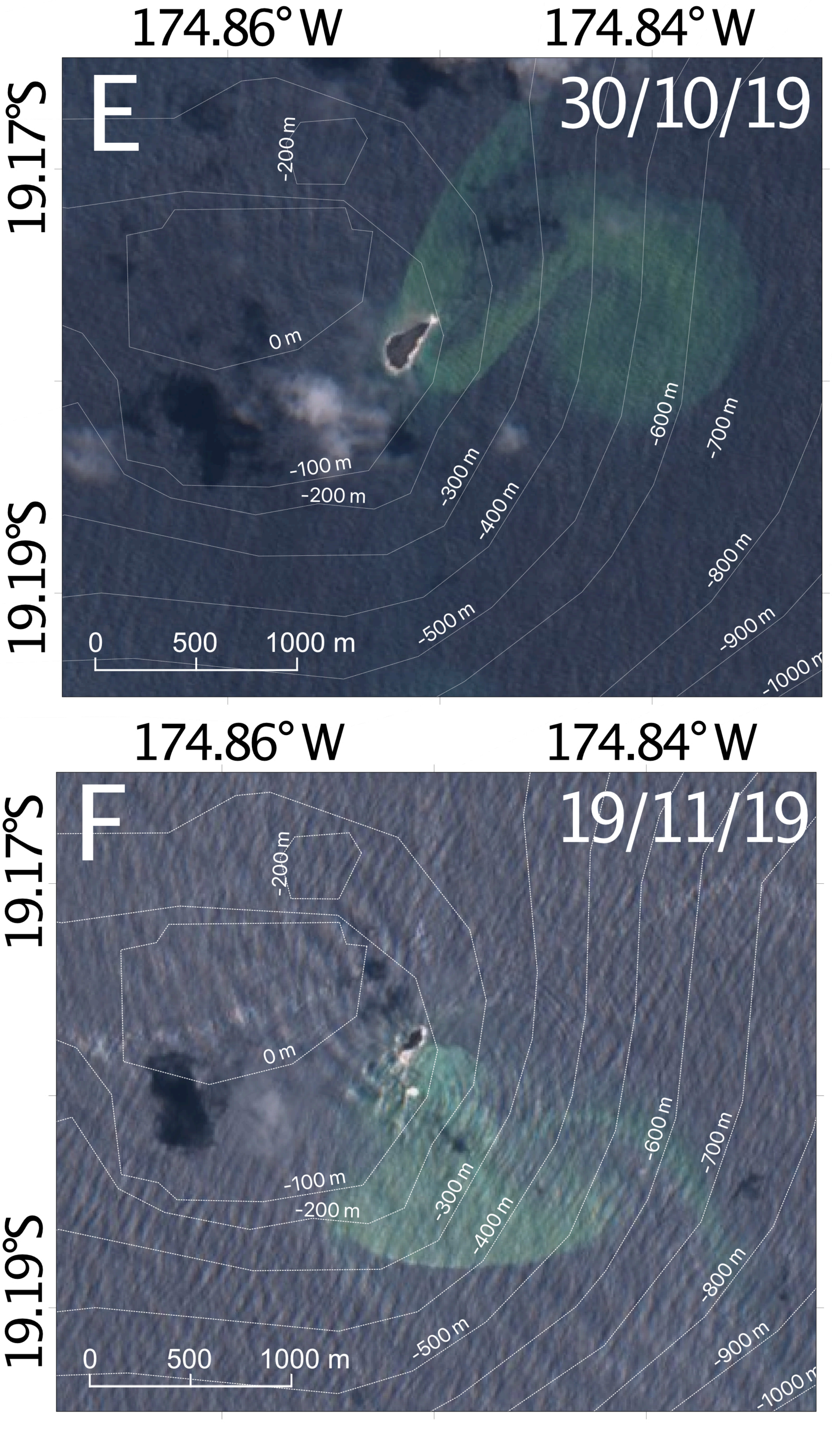

174.86° W
174.84° W
19.17°S
19.19°S
E
30/10/19
-200 m
0 m
-100 m
-200 m
-300 m
-400 m
-500 m
-600 m
-700 m
-800 m
-900 m
-1000 m
0
500
1000 m
174.86° W
174.84° W
19.17°S
19.19°S
F
19/11/19
-200 m
0 m
-100 m
-200 m
-300 m
-400 m
-500 m
-600 m
-700 m
-800 m
-900 m
-1000 m
0
500
1000 m

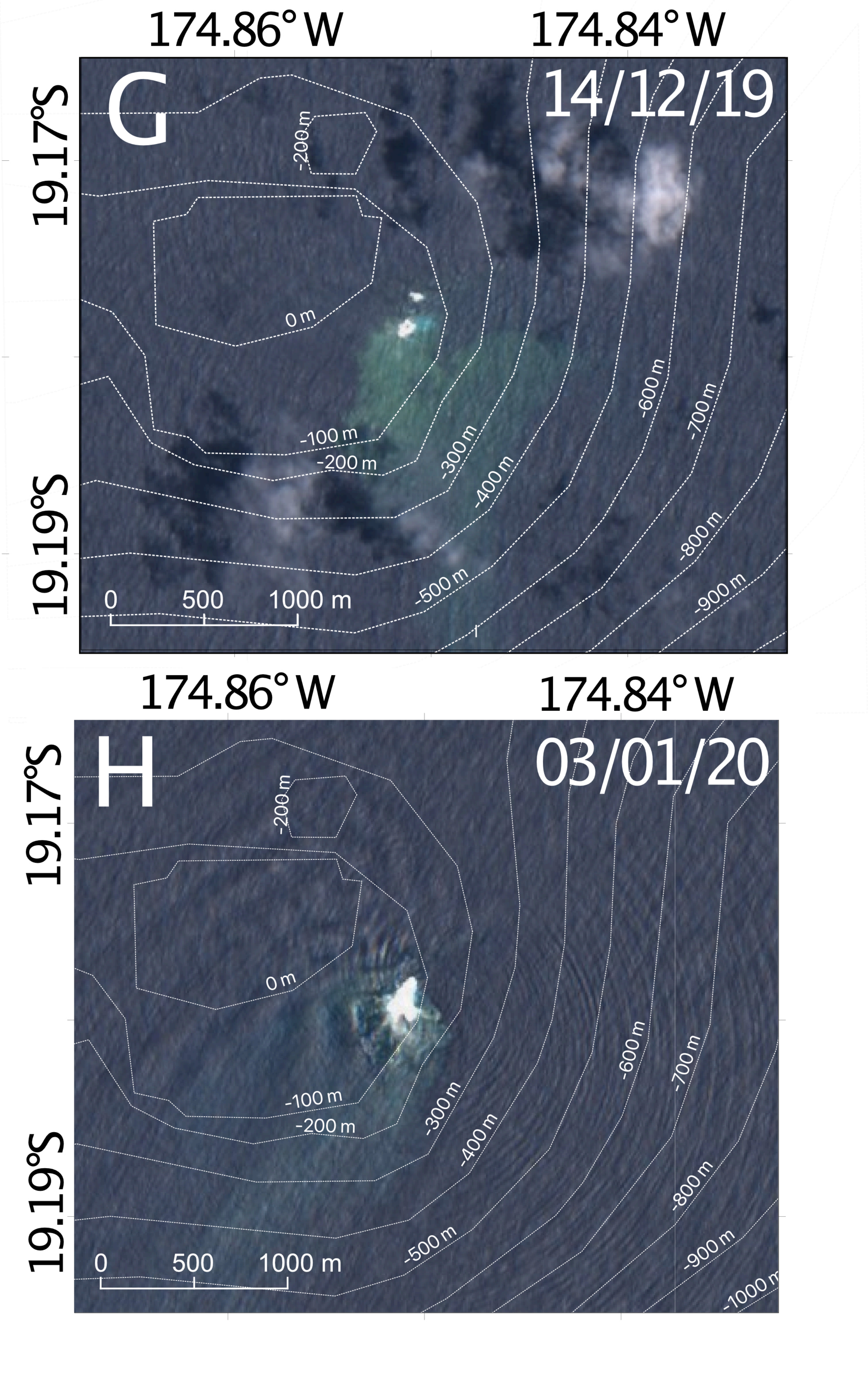

174.86° W
174.84° W
19.17°S
19.19°S
G
14/12/19
0 m
-100 m
-200 m
-200 m
-300 m
-400 m
-500 m
-600 m
-700 m
-800 m
-900 m
0
500
1000 m
174.86° W
174.84° W
19.17°S
19.19°S
H
03/01/20
0 m
-100 m
-200 m
-200 m
-300 m
-400 m
-500 m
-600 m
-700 m
-800 m
-900 m
-1000 m
0
500
1000 m

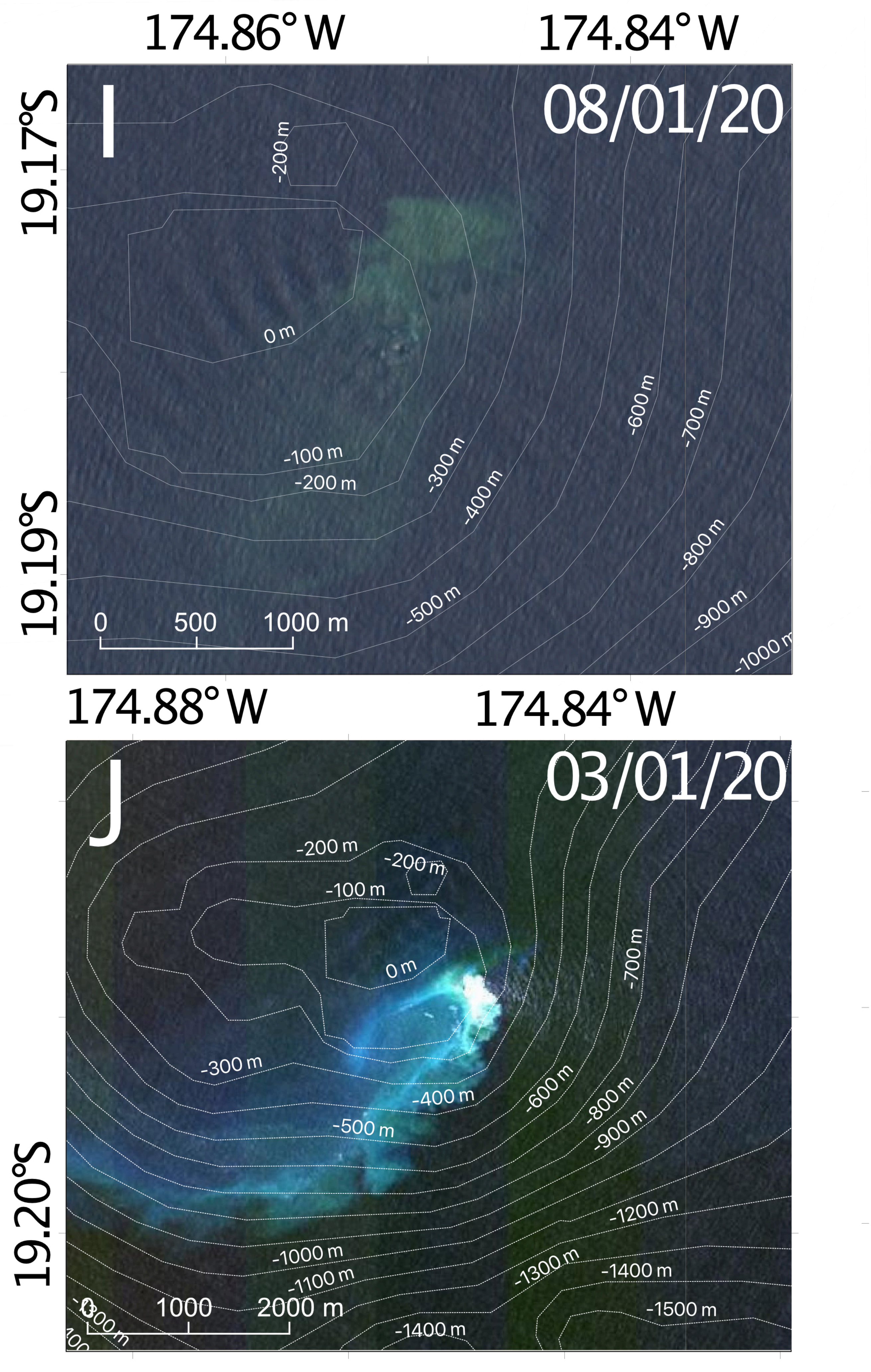
174.86° W
174.84° W
19.17°S
19.19°S
I
08/01/20
-200 m
0 m
-100 m
-200 m
-300 m
-400 m
-500 m
-600 m
-700 m
-800 m
-900 m
-1000 m
0
500
1000 m
174.88° W
174.84° W
19.20°S
J
03/01/20
-200 m
-200 m
-100 m
0 m
-300 m
-400 m
-500 m
-600 m
-700 m
-800 m
-900 m
-1000 m
-1100 m
-1200 m
-1300 m
-1400 m
-1400 m
-1500 m
0
1000
2000 m

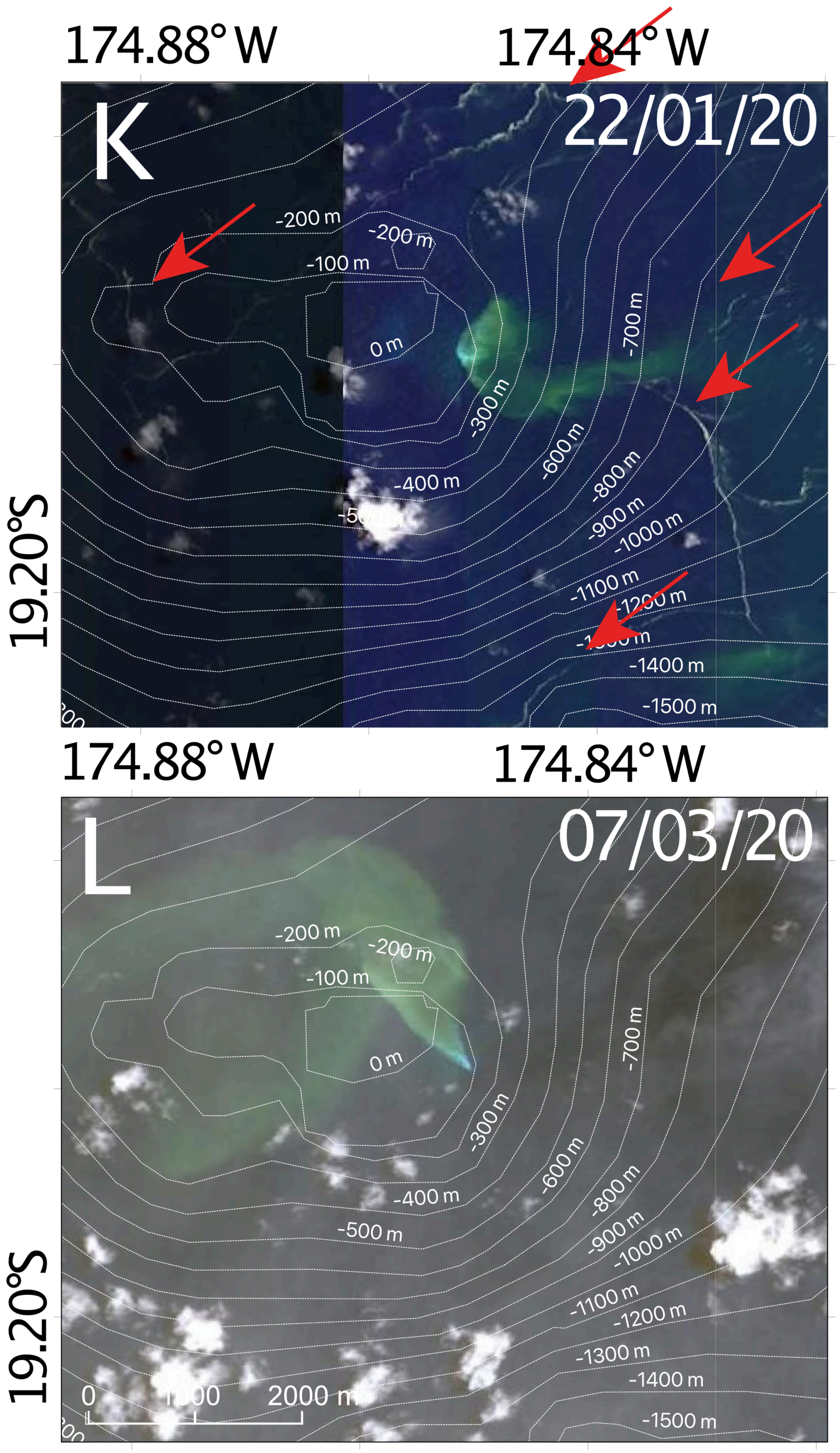
174.88° W
174.84° W
K
22/01/20
-200 m
-200 m
-100 m
0 m
-300 m
-400 m
-600 m
-700 m
-800 m
-900 m
-1000 m
-1100 m
-1400 m
-1500 m
19.20°S
174.88° W
174.84° W
L
07/03/20
-200 m
-200 m
-100 m
0 m
-300 m
-400 m
-500 m
-600 m
-700 m
-800 m
-900 m
-1000 m
-1100 m
-1200 m
-1300 m
-1400 m
-1500 m
19.20°S
0
2000 m
